# EEG Source Imaging Guided by Spatiotemporal Specific fMRI: Toward an Understanding of Dynamic Cognitive Processes

**DOI:** 10.1155/2016/4182483

**Published:** 2016-10-10

**Authors:** Thinh Nguyen, Thomas Potter, Trac Nguyen, Christof Karmonik, Robert Grossman, Yingchun Zhang

**Affiliations:** ^1^Department of Biomedical Engineering, Cullen College of Engineering, University of Houston, Houston, TX 77204, USA; ^2^MRI Core, Houston Methodist Research Institute, Houston, TX 77030, USA; ^3^Department of Neurosurgery, Houston Methodist Hospital and Research Institute, Houston, TX 77030, USA; ^4^Guangdong Provincial Work Injury Rehabilitation Center, Guangzhou 510000, China

## Abstract

Understanding the mechanism of neuroplasticity is the first step in treating neuromuscular system impairments with cognitive rehabilitation approaches. To characterize the dynamics of the neural networks and the underlying neuroplasticity of the central motor system, neuroimaging tools with high spatial and temporal accuracy are desirable. EEG and fMRI stand among the most popular noninvasive neuroimaging modalities with complementary features, yet achieving both high spatial and temporal accuracy remains a challenge. A novel multimodal EEG/fMRI integration method was developed in this study to achieve high spatiotemporal accuracy by employing the most probable fMRI spatial subsets to guide EEG source localization in a time-variant fashion. In comparison with the traditional fMRI constrained EEG source imaging method in a visual/motor activation task study, the proposed method demonstrated superior localization accuracy with lower variation and identified neural activity patterns that agreed well with previous studies. This spatiotemporal fMRI constrained source imaging method was then implemented in a “sequential multievent-related potential” paradigm where motor activation is evoked by emotion-related visual stimuli. Results demonstrate that the proposed method can be used as a powerful neuroimaging tool to unveil the dynamics and neural networks associated with the central motor system, providing insights into neuroplasticity modulation mechanism.

## 1. Introduction

Cognitive rehabilitation often involves and relies on the modulation of neuroplasticity to improve motor function. However, the neuroplasticity mechanisms associated with the central motor system remain poorly understood. While the brain regions involved in motor execution are well characterized, the detailed dynamics of these neural events and the underlying neural network changes still remain unclear. Neuroplasticity, the ability of the brain to alter neuronal connections, can be expressed on both anatomical and functional levels. The anatomical aspects of plasticity can be captured directly via structural imaging (e.g., MRI, DTI) [1, 2]. Accordingly, functional markers (delta waves, nonrapid eye movements, event related synchronization/desynchronization, etc.) of neural plasticity have also been identified in a variety of conditions [3–5]. Properly investigating these markers, however, remains a major challenge since it requires advanced imaging modalities with both high temporal and spatial resolution. Thoroughly characterizing the dynamic processes of the neuromuscular system, in both healthy and diseased brains, is the first step toward understanding the plastic changes that occur during neural diseases, injuries, and the treatment outcomes of different rehabilitation programs. Multiple noninvasive neuroimaging methods have been developed to achieve this goal, among which functional Magnetic Resonance Imaging (fMRI) and Electroencephalography (EEG) currently stand as prevailing techniques.

fMRI comprises one of the primary methods for observing neural activity. As an extension of anatomical MRI, fMRI utilizes a series of strong magnetic fields in concert with native local inhomogeneities within the body to create a Blood Oxygen Level Dependent (BOLD) contrast that identifies regions with significantly different concentrations of oxygenated blood [6]. This serves as an indirect measure of underlying neuronal activity—the high metabolic demand of active brain regions requires an influx of oxygen-rich blood [7], increasing the intensity of voxels where activity can be observed [8]. Typical analysis for this convolves the detected timescale peaks with a hemodynamic response. The method continues by utilizing a general linear model that treats different conditions, motion parameters, and polynomial baselines as regressors to generate a map of significantly activated voxels [9, 10], ultimately creating a static but spatially accurate depiction of cortical BOLD fluctuations and, by extension, the underlying neural activity.

Beyond simple statistical tests, further analysis can be performed on fMRI data to elucidate networks of functional activity across the disparate brain regions. Instead of treating conditions as regressors, measures of correlation are calculated from the time courses of the BOLD signals of voxels and regions to identify highly correlated regions and the statistical dependencies between them [11, 12]. Correlations such as these can be applied in both the time and frequency domains and open the door for further analysis under graph theory, which creates a mathematical representation of functional networks by modeling voxels or regions as “nodes” and the connections between them as “edges.” A variety of potential measures are available under this graph theory to describe the networks within the brain and their arrangement [13], each of which has shown use in different conditions or diseases, including the analysis of emotion processing [9, 14–20]. Together, these methods provide robust and varied methods for examining functional MRI data and the activity it reveals.

Electroencephalography (EEG), in contrast to fMRI, represents a method for directly detecting and recording electrical signals associated with neural activity from scalp electrodes. As signals are transduced from neuron to neuron, the postsynaptic potentials that result from neurotransmitter detection create electrical activity which, while individually weak, sums to produce larger voltage potentials [21]. With a series of electrodes measured against a reference at rapid sampling rate, EEG is able to generate temporally accurate measurements of these voltage differences on the scalp [22]. Unfortunately, the transduction of these signals through the brain, cerebrospinal fluid, skull, and scalp blurs the results, limiting the effective spatial resolution of the modality [23]. Source imaging methods have been developed to alleviate this limitation by modeling the intermediate layers and their conductivities and backwards calculating the origin of the sources [24]. However, this still poses a technical challenge, as the “inverse problem” in the source imaging methods is ill-posed: the number of variables vastly outnumber the available data [25]. Common source imaging analysis makes use of a pseudoinversion to circumvent this and results with focal spatial localization can be achieved by further minimizing a net current vector [26]. This solution, however, relies on a maximized likelihood instead of clear results and subsequently suffers from complex calculation and spatial imprecision.

EEG and fMRI can be viewed as complimentary imaging modalities. fMRI alone is limited as hemodynamic signals that only provide an indirect measure of the neuronal activity with a poor temporal resolution (second level). In contrast, EEG directly measures dynamic electrophysiological activity of the brain with a very high temporal resolution (millisecond level), but poor spatial resolution. These properties have led to multimodal approaches seeking to optimize the favorable properties from each [27]. Simultaneous EEG and fMRI allow the excellent temporal resolution of EEG to be combined with the high spatial accuracy of fMRI to overcome the limitations associated with unimodal fMRI or EEG.

Asymmetrical integration methods make use of EEG to inform fMRI [27] or fMRI BOLD maps as constraints to guide EEG localization [28, 29]. These methods, however, are subject to localization mismatch and bias [30]. Traditional EEG/fMRI integration and connectivity analysis starts with fMRI-informed EEG source localization. Usually, an fMRI-derived BOLD activation map is used to construct spatial constraints on the source space in the form of a source covariance matrix, whereas active sources not present in the fMRI are penalized [28, 29, 31]. The performance of this approach also relies on the accuracy of EEG source analysis, which could be spatially biased due to the use of fMRI BOLD activation map as “hard” constraints, in the sense that fMRI-derived spatial information is considered an absolute truth in guiding the EEG source analysis. As such, EEG source reconstruction could be strongly biased in the event of an EEG-fMRI mismatch [30] (due to neurovascular decoupling, signal detection failure, etc.).

The Bayesian framework has recently been developed [32–35] to perform fMRI-informed EEG source imaging utilizing fMRI information as “soft” constraints. In particular, two-level hierarchical empirical Bayesian models are used to model the EEG inverse problem, where parameters at the first level represent unknown source activity and the 2nd-level parameters (hyperparameters) model the prior distribution of the 1st-level parameters (equivalent to source covariance matrix). Model inversion is done using an Expectation Maximization (EM) technique that estimates the hyperparameters that would maximize the model evidence and, in turn, estimate the parameters of interest—EEG source activity (see [33, 36] for details on Bayesian model inversion scheme). These methods incorporate fMRI information as “soft” constraints, in which the fMRI-active map is modeled as a prior and its relative weighting is estimated via the hyperparameters. The hierarchical empirical Bayesian framework allows the fMRI information to be modeled as a weighted sum of multiple submaps, representing multiple priors, controlled by corresponding weighting hyperparameters, with values to be estimated. However, the issue of temporal mismatch between EEG-fMRI still persists.

In this study, we propose a spatiotemporal fMRI constrained EEG source imaging approach to address the issue of temporal mismatch between EEG-fMRI by calculating the optimal subset of fMRI priors (in terms of model evidence) based on a hierarchical Bayesian model. fMRI priors were computed in a data-driven manner from particular windows of interest in the EEG data, leading to time-variant fMRI constraints. The proposed approach utilizes the high temporal resolution nature of EEG to compute a current density mapping of the cortical activity, informed by the high spatial resolution of fMRI in a time-variant, spatially selective manner, to accurately image dynamic neural activity. The high spatiotemporal features of this method then make it particularly desirable for studying the central motor system and functional aspects of plasticity as they relate to cognitive rehabilitation.

## 2. Methodology

### 2.1. Data Model

Considering a linear model of EEG data *Y* ∈ *ℝ* ^*m*×*d*^ over *m* channels and *d* measurement samples:

(1) $$\begin{array}{c} Y=GJ+\varepsilon ,\;\varepsilon \sim N\left(0,C\right),\;\;J\sim N\left(0,R\right), \end{array}$$

where *G* ∈ *ℝ* ^*m*×*s*^ represents the lead field matrix and *J* ∈ *ℝ* ^*s*×*d*^ represents the unknown source activity of *d* dipole sources in the source space. *ε* represents the noise component in the sensor space with its noise covariance matrix *C*. *R* represents the source covariance matrix. The current density *J* can be reconstructed according to (2) by applying the *L*_2_-norm inversion scheme [26] to the model above.

(2) $$\begin{array}{c} J=RG^{T}{\left(\;GRG^{T}+\lambda^{C}C\;\right)}^{-1}Y. \end{array}$$

The regularization parameter, *λ* ^*C*^, could be seen as the trade-off between model accuracy and complexity and is traditionally determined using the L-curve method. The source covariance matrix *R* represents prior knowledge about the distribution of *J*. *R* will be an identity matrix, *I*, if no prior assumption has been made to the distribution of *J* and *R* will be constructed according to the fMRI activation map in a EEG/fMRI integration approach [28]. Thus, the source space *J* is subjected to a prior spatial constraint based on the active voxels from the BOLD mapping. However, imposing such time-invariant fMRI constraints might not be appropriate for all time instances.

### 2.2. Spatiotemporal fMRI Constrained EEG Source Imaging

#### 2.2.1. fMRI Data Analysis

The classical general linear model (GLM) is employed for statistical analysis of preprocessed fMRI data, and a map of the voxels that show statistically significant activity is achieved when contrasted between two or more conditions (e.g., task versus baseline). Voxel values in the fMRI map below a certain *p* value threshold (*p* < 0.05) are omitted, to ensure that only statistically significant voxels are used as constraints for the source imaging routine.

In this study, the fMRI activation map is further divided into multiple submaps based on clusters of neighboring locations or cortical functional regions for spatial flexibility in applying the fMRI information as a constraint.

#### 2.2.2. The Source Covariance Matrix *R*

In our current framework, we employ the construction of *R* as follows:

(3) $$\begin{array}{c} R=\overset{N}{\underset{i=1}{\sum }}\lambda_{i}^{R}Q_{i}. \end{array}$$

*R* is defined by the sum of *N* covariance components *Q* = {*Q*_1_,…, *Q*_*N*_}, weighted by an unknown hyperparameter *λ* ^*R*^. Each covariance component, *Q*_*i*_ = *q*_*i*_ *q*_*i*_^*T*^, is formed from a subset *q*_*i*_ of the fMRI map as explained above.

#### 2.2.3. Space Time Specific fMRI Priors

Given EEG data *Y*, an optimized weighted combination of these *N* priors via their respective *λ*_*i*_^*R*^ is determined by maximizing the log model evidence ln⁡*p*(*Y*∣*λ*), where *λ* = {*λ* ^*C*^, *λ* ^*R*^}.

(4) ln⁡pY ∣ λ=ln⁡∫pY,J ∣ λdJ,ln⁡pY ∣ λ=F+DqJ||pJ ∣ Y,λ,

where *F* is the variational free energy and a lower bound on the evidence. Maximizing this boundary would minimize the Kullback-Leibler divergence *D*(*q*(*J*)||*p*(*J*∣*Y*, *λ*)), so that the free energy approximates the log-evidence, *F* ≈ ln⁡*p*(*Y*∣*λ*).

*F* can then be calculated as

(5) F=−12trΣμλ−1YYT−12ln⁡Σμλ−12ln⁡2π+12ln⁡Σλ−12μλ−ηTΠμλ−η,

where Σ(*μ* ^*λ*^) = *GRG* ^*T*^ + *C* and the conditional density of the hyperparameters is *q*(*λ*) = *N*(*μ* ^*λ*^, Σ^*λ*^) with its prior *p*(*λ*) = *N*(*η*, Π^−1^), where *η* = −32, Π = 256 (for a detailed derivation and discussion of the free energy *F*, see [33, 36]).

Given this model, *F* is equivalent to a ReML objective function and can be maximized using a classical ReML algorithm. The maximization of *F* yields (*μ* ^*λ*^, Σ^*λ*^) and a model evidence which could further be used for model comparison. When *λ* = *μ* ^*λ*^, an optimal weighted combination of fMRI priors {*q*_1_, *q*_2_,…, *q*_*N*_} is determined and the corresponding source dynamics *J* can be solved for the given time window of interest.

#### 2.2.4. EEG Source Imaging

**Figure 1:**
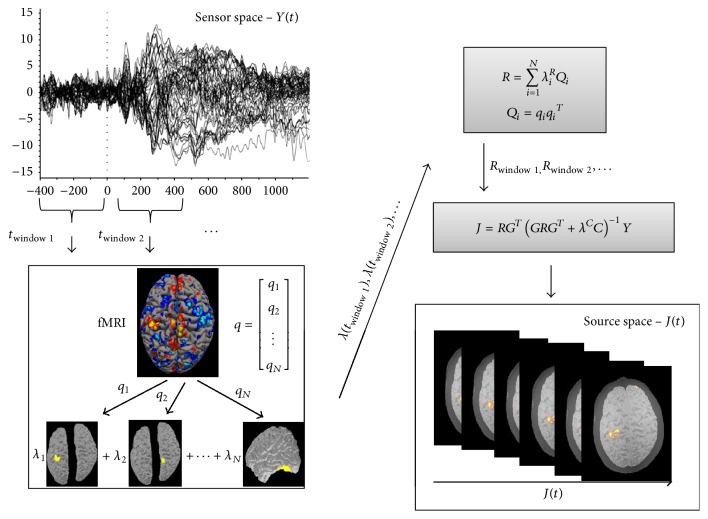
Schematic of the spatiotemporal fMRI constraints on EEG source imaging.

EEG data is divided into different time windows. EEG information in each time window is then used to estimate the hyperparameter *λ* ^*R*^ by estimating the model evidence ln⁡*p*(*Y*∣*λ*) via maximizing the variational free energy (4). The source covariance matrix *R* is determined from the estimated hyperparameter *λ* ^*R*^ using (3) for each time window. The dynamic activity of the source *J*, constrained by *R*, is calculated in (2).  Figure 1 shows a schematic of the approach described above. Instead of applying a static fMRI map as a constraint for all time instances, multiple subsets of the fMRI information are employed as spatial priors in a weighted manner and the weighting factors are determined by the EEG data in each specific time period. As such, EEG source imaging constrained by fMRI in spatiotemporal specific fashion is achieved.

### 2.3. Experimental Setup and Data Processing

#### 2.3.1. The Paradigm

**Figure 2:**
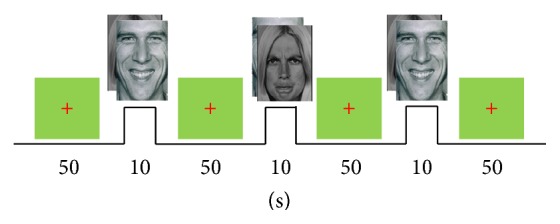
Experimental paradigm—rest state: subjects were shown a green background (50 seconds); active state: display of face image (two categories: unpleasant and pleasant); subjects are to squeeze a ball with right hand for 10 seconds if image was perceived as unpleasant.

EEG and fMRI data were acquired from three healthy male subjects (age 22 to 26 years) participating in a visual stimulus/motor response experiment under a research protocol approved by the local ethic committee. The paradigm consisted of a series of visual stimuli, each belonging to one of two categories: pleasant faces and unpleasant faces as illustrated in Figure 2. In each trial, a 50-second green screen baseline was first shown, followed by a categorically randomized 10-second visual stimulus. The subject was instructed to squeeze a rubber ball with his right hand for the entire duration the stimulus image was shown if he perceived the presented face as unpleasant, while remaining at rest if the image was perceived as pleasant. The fMRI data from 5 pleasant and 5 unpleasant trials were collected while the EEG data from 40 pleasant and 40 unpleasant trials were collected outside the MRI room using the same experimental paradigm.

#### 2.3.2. Anatomical and Functional MRI Data Acquisition

EEG and fMRI scans were performed during different sessions for each subject. fMRI data acquisition (Philips Ingenia 3.0T) was performed using gradient echo Echo-Planar Imaging, with repetition time (TR) of 1500 ms, echo time (TE) of 35 ms, and voxel size of 3 × 3 × 5 mm. fMRI data underwent a conventional fMRI preprocessing pipeline: realignment, slice timing correction, motion correction, coregistration, segmentation, normalization, and spatial smoothing (FWHM of 3 mm) were applied. The structural MRI for each subject was also obtained for fMRI coregistration and subject-specific head model generation. A T1-weighted MRI image was acquired using gradient echo, with TR = 8.1 ms, TE = 3.7, and voxel size 0.9 × 0.9 × 1 mm.

T1-weighted structural MRI images for each subject underwent full reconstruction procedure using the Freesurfer image analysis suite (publicly available at: http://surfer.nmr.mgh.harvard.edu/), resulting in generation of a high-definition cortical layer and brain-skull-skin layer. The high-density cortical layer mesh was downsampled to ~10,000 vertices per hemisphere and used as the source space, where each vertex location corresponds to a dipole source oriented perpendicular to the surface. A lead field matrix *G* was computed via a forward calculation using the cortical source space, a 3-layer head model, and 64-channel electrode locations coregistered to the model.

#### 2.3.3. fMRI-EEG Data Acquisition and Processing

fMRI data analysis was performed using the Freesurfer software suite. Spatial blurring was applied using a Gaussian kernel with a 3 mm full width at half maximum (fwhm). The hemodynamic response was modeled using a 0th derivative canonical SPM hemodynamic response function. The two experimental conditions and six motion parameters were used as regressors in the general linear model (GLM) for the statistical analysis of the fMRI data, with a 2nd-order nuisance regressor included to correct for noise. Statistical *t*-maps contrasting experimental conditions against baseline were generated for each subject. Correction for multiple comparisons was performed by controlling the Family Wise Error Rate (FWER). A cluster-based FWE-corrected *p* value of 0.05 was set, such that only clusters large enough to surpass the *p* < 0.05 threshold were considered activated. Individual fMRI maps were translated to corresponding subject-specific cortical models and a 4-voxel cluster-extent threshold was applied to account for any erroneous voxel activity resulting from the translation process. EEG recordings were performed with a sampling rate of 5 kHz using a 64-channel EEG recording system (Brain Products, Germany). EEG data was preprocessed using BrainVision Analyzer 2.0 (Brain Products, Germany). Rereferencing to a linked mastoid and a band pass filter from 0.5 Hz to 50 Hz were employed along with a notch filter at 60 Hz. EKG artifacts were removed by means of template subtraction; ocular artifacts and movement-related artifacts were further removed by independent component analysis (ICA). The EEG data with unexpected artifacts and/or noise were removed, resulting in an EEG data set consisting of 36 pleasant trials and 37 unpleasant trials. Single-trial EEG data were employed and segmented from 400 ms prior to the visual stimulus onset (at 0 ms) to 1200 ms after the onset. Ball squeezing occurred at an average time of 600 ms (ranging from 500 ms to 800 ms) after visual stimulus onset. Each epoch was baseline corrected using a baseline measured from −400 ms to 0 ms. EEG data were segmented evenly to yield 40 individual time windows in this study. A window length of 40 ms was employed to maintain high specificity and accommodate the timeline of anticipated ERPs associated with the experimental paradigm with a minimal risk of clipping evoked potentials (VEP, P300, MEP, etc.) [37, 38].

## 3. Results

### 3.1. fMRI

**Figure 3:**
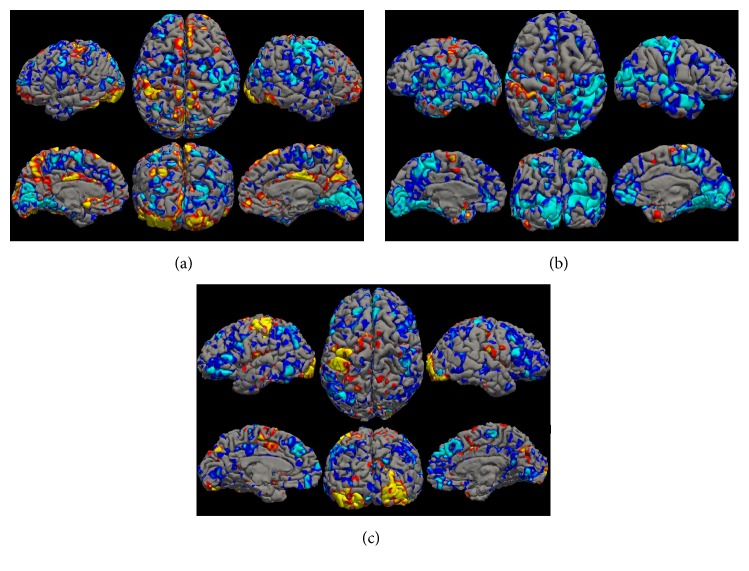
Unpleasant-face response versus baseline fMRI activation map showing regions of high BOLD signal for subjects #1, #2, and #3 in (a), (b), and (c), respectively.

The fMRI BOLD activation map shows statistically significant regions of cortical activity during the visually evoked and motor responses of the participant responding to the unpleasant-face stimulus (see Figure 3). The dominant activated regions were found to be in the bilateral visual cortices, left motor cortex, fusiform face area, supplementary motor area, and posterior cingulate cortex.

### 3.2. Model Comparison

**Figure 4:**
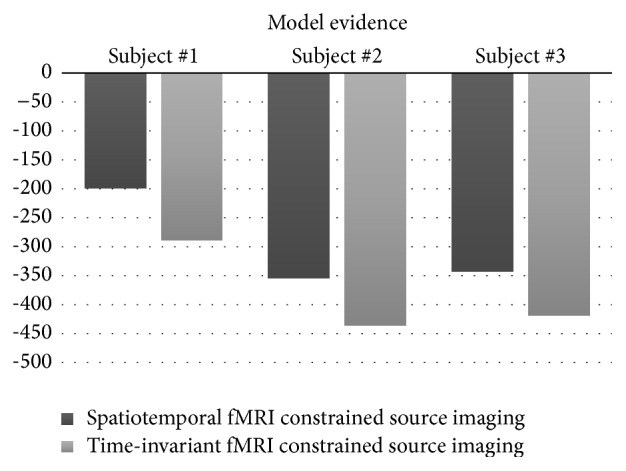
Model comparison between two methods: time-invariant fMRI constraints and spatiotemporal fMRI constraints source imaging for three subjects. Model comparison, given in terms of log model evidence ln⁡*p*(*Y* | *λ*), serves as a relative metric to compare performance of different data models, with higher value depicting better model.

The model evidence *p*(*Y*∣*λ*) describes how well a given model can explain the measured data; the absolute value itself is arbitrary and should only be interpreted as a relative metric to compare the performance of different models. The average log-evidence ln⁡*p*(*Y*∣*λ*) was computed across the time course of each epoch, comparing two models: the spatial and temporal variant fMRI-based prior model and the time-invariant fMRI constraints model. The results suggest better performance using the spatiotemporal variant fMRI model consistently across all three subjects as shown in Figure 4.

### 3.3. Validation

Two source imaging methods, that is, the traditional time-invariant fMRI constraints source imaging and spatiotemporal fMRI constrained EEG source imaging methods, were implemented and compared in terms of the performance in characterizing source dynamics in the brain. Source analysis was performed in a single-trial manner. For each trial, EEG data was divided into multiple time windows of interest and the weights for source priors were determined. Dynamic source activity for each time window was then computed using the calculated weights (as described in Section 2).

**Figure 5:**
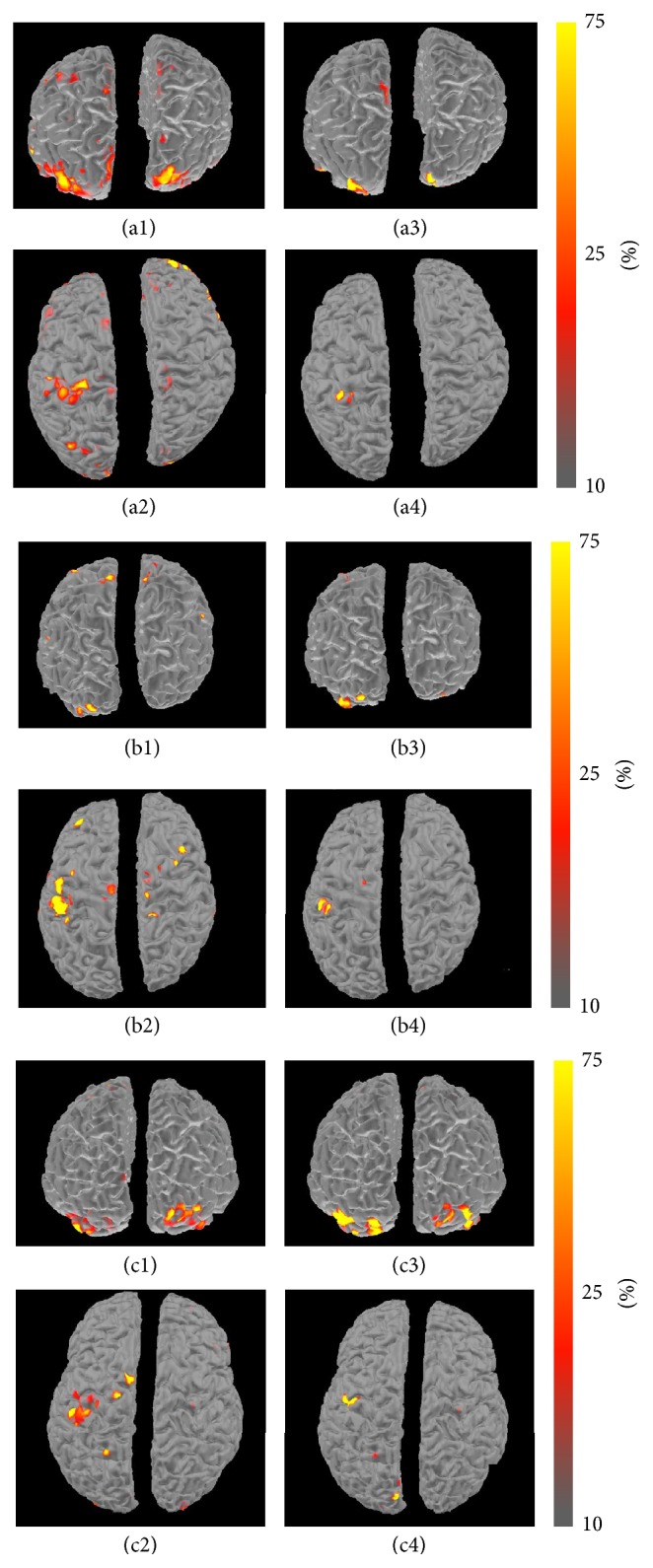
Source imaging results comparing two EEG/fMRI integration approaches: time-invariant fMRI constraints ((a1), (a2); (b1), (b2); (c1), (c2)) and spatiotemporal fMRI constraints ((a3), (a4); (b3), (b4); (c3), (c4)) at two time instances to demonstrate visual activation ((a1), (a3); (b1), (b3); (c1), (c3)) and motor activation ((a2), (a4); (b2), (b4); (c2), (c4)). Source activity shown is color coded as a percentage of its maximum.

Figure 5 compares the neural activity at two time points of interest, as calculated by classical time-invariant algorithm (left; subfigures (1) and (2)) and the new spatiotemporal algorithm (right; subfigures (3) and (4)). For the subjects and the trials analyzed, both methods showed the increase of current density consistently in the bilateral visual cortices and left motor cortex. For subject #1, visual cortex activation was found at 260 ms (Figures 5(a1) and 5(a3)) with motor cortex activation at 610 ms (Figures 5(a2) and 5(a4)), and the subject response time was 592 ms. For subject #2, visual cortex activation was found at 125 ms (Figures 5(b1) and 5(b3)) and motor cortex activation was seen at 680 ms (Figures 5(b2) and 5(b4)), with a subject response time of 612 ms. Similarly, activity at visual cortex on subject #3 was found at 110 ms (Figures 5(c1) and 5(c3)) and the motor cortex activity peaked at 800 ms (subject #3 had a response time of 704 ms). However, it can be observed that the source activity reconstructed using the proposed method yielded sparser, more localized results when compared to the conventional time-invariant fMRI-informed source imaging method.

**Figure 6:**
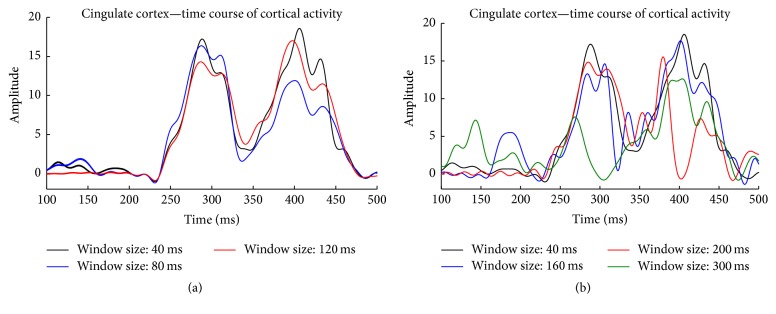
Cortical activity of the cingulate cortex calculated with different window sizes. Results with smaller window sizes show a great similarity (correlation *R* > 0.95) in (a); and results with larger window sizes show a greater disparity (correlation *R* < 0.7) in (b).

The stability of the proposed method was investigated by examining how the reconstructed source activity changes with changes in the window size.  Figure 6 shows the change in the activity time course for a source in the cingulate cortex when reconstructed with different window sizes. For window sizes below 120 ms, EEG source localization results showed minimal changes, as shown in Figure 6(a). The results began to lose detail and specificity, however, when window sizes increased above 120 ms (Figure 6(b)). While there is no limitation regarding the minimum window size used in the proposed algorithm, reducing the window size below 40 ms is not necessary as the changes in the estimated hyperparameter (*λR*) became very small, yet the cost of computational effort increases dramatically.

### 3.4. Transition Period

**Figure 7:**
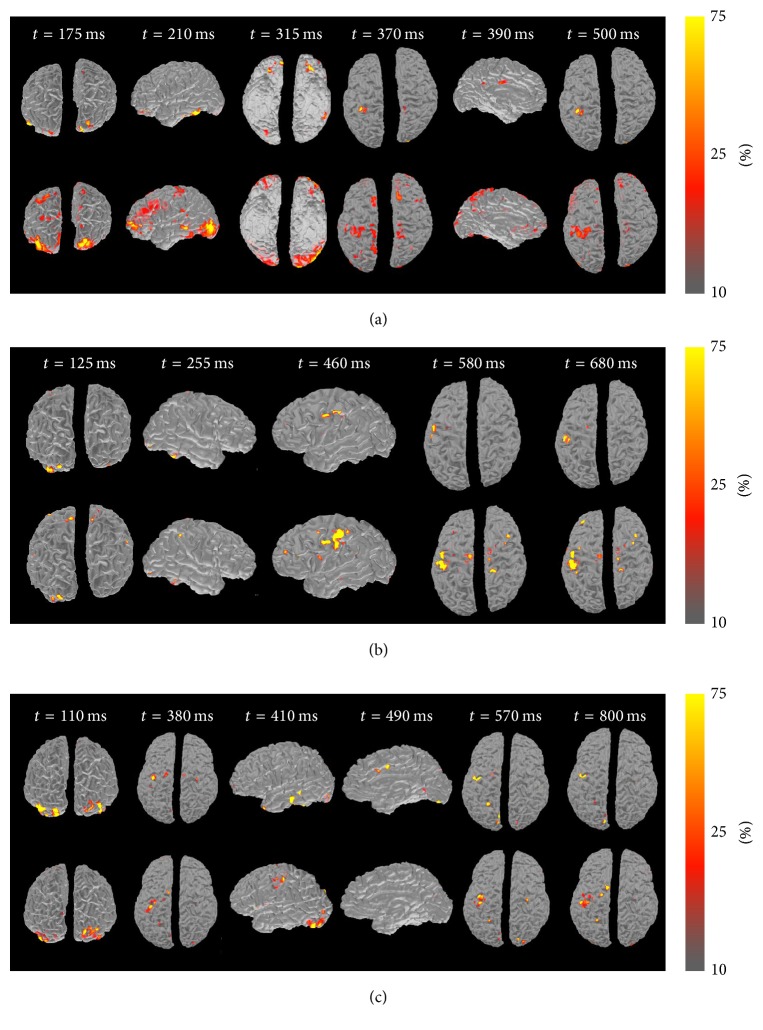
Transition period source imaging results for subjects #1, 2, and 3 in (a), (b), and (c), respectively. In each subfigure, top panel represents source imaging results using spatiotemporal fMRI constrained method, while bottom panel shows time-invariant fMRI constrained method. Results highlight cortical activity at different time instances during the period of time transitioning between a visual input to subject's response via motor output. Time stamps shown are with respect to the visual stimulus onset timing (at *t* = 0 ms). Source activity shown is color coded as a percentage of its maximum.

Examining the results of our algorithm in the transition period (Figure 7), several trends can be ascertained. Subjects showed an early peak of visual activation, followed by activation in the cingulate cortex and fusiform face area. Subsequently, activation can be observed in the motor cortex, followed by second peak of activity. Interestingly, the early activity in the motor cortex can be observed well before any physical motor activity is initiated. Squeezing of the ball is related to the second peak of the motor cortex activation. When comparing the specific source imaging with the time-invariant counterpart, the new algorithm consistently results in more precise and focused results. Areas of moderate activation (orange) are greatly reduced, creating results that are both sparse and high in contrast.

## 4. Discussion

A new method has been developed in this study to utilize a time-variant fMRI constraint in conjunction with EEG source imaging to produce a more precise and focused depiction of neural activity without amplifying erroneous signals. The Bayesian framework has been recently developed [32–35] to improve fMRI-informed EEG source imaging results by utilizing “soft” fMRI constraints, but the issue of temporal mismatch between EEG-fMRI still persists. This is especially problematic for neurological studies that explore the dynamic brain activity during the rapid transition periods between cognitive tasks—transition periods that may contain valuable information regarding the presentation of neural plasticity markers and how stimulus processing changes under various conditions. Furthermore, many paradigms do not allow for EEG data to be averaged over multiple occurrences or epochs, in cases where responses change over time (habituation) or are not time-locked (i.e., latency between stimulus and response). Difficulty in these cases may be further accentuated by the relatively static nature of utilized fMRI data. While EEG signals vary in a time dependent nature, BOLD signals remain static regardless of condition or timing and could potentially amplify irrelevant or erroneous sources in a multimodal framework. The proposed spatiotemporal fMRI constrained EEG source imaging approach utilizes the EEG data in a selected time window to determine the best-fit source prior from the fMRI BOLD activation map. The resulting fMRI priors are in turn utilized in fMRI-informed EEG source localization in order to solve the timing mismatch between EEG and fMRI.

The proposed approach was implemented and tested in an EEG/fMRI study on motor activation in response to emotionally evocative visual stimuli. The processed windowed EEG signals were analyzed to select the temporally relevant areas of fMRI activity, which were used to inform EEG source localization calculation. The results were compared against traditional fMRI-informed EEG approaches to demonstrate the spatiotemporal variant fMRI priors feature as well as the performance of the developed method.

The initial results from fMRI alone support our hypothesis that the task would involve visual stimulation, facial recognition, decision making, and motor responses, highlighting these as areas of interest for the new algorithm. The fMRI alone, however, still faces difficulty as no dynamic neural pathways relating to these events could be inferred from the BOLD activation map. Similarly, though EEG has the requisite temporal resolution to examine any potential pathways, the need for localization algorithms limits its use.

The new algorithm was compared to the traditional time-invariant fMRI constrained EEG source imaging method in a visual-motor response paradigm. The results showed similar areas of activation in the motor cortex, visual cortex, fusiform face area, supplementary motor area, and posterior cingulate cortex. While similar areas were highlighted under both conditions, the new algorithm produced results that are more spatially precise, with fewer areas showing moderate or low amplitude results. In contrast with the precise results from spatiotemporal fMRI constrained EEG source imaging method, the dispersed source imaging results seen in the traditional method were likely caused by the spatial bias of using time-invariant fMRI constraints, as the same fMRI spatial information might not be valid for all time instances. It should further be noted that while the same general areas can be observed, the exact location of activity within these regions may be slightly shifted under the time-variant constraints (Figure 5).

With reference to the performed test, results are consistent with areas expected to be activated in a face-based visual-motor paradigm. Activation could first be observed in the visual cortex and fusiform face area at 100–175 ms after stimulus onset. As faces were utilized as the primary stimuli, activation in these regions is expected and aligns with findings in current literature [39, 40]. Following the visual activation, activity can be observed within the hand regions of the premotor and motor cortices (370–460 ms). While these sources do appear in predictable regions, their timing makes them a significant point of interest: motor response for the subjects is not observed until 500–570 ms after stimulus presentation. The hand region also shows activity at these times, and the early peak in activity may represent a previously unidentified premotor activation. Following this premotor wave, activity can be seen in the cingulate cortex, which is often associated with the emotional processing of the happy and sad stimuli and again fits with a previous report [41]. Finally, strong activation was observed in the motor cortex as motor activity took place. The results achieved in the performed task are overall consistent with our expectations. Aside from being spatially consistent, the time course of activation also indicates a dynamic neural pathway that is integral to the stimulus detection and response, starting in the visual cortex and proceeding to the motor cortex.

It is a grand challenge in the field to accurately and noninvasively detect and localize neural activity, let alone the transient markers associated with cortical plasticity. Though many technologies have been developed to accomplish this task, there has yet to be a complete solution that allows for both favorable temporal and spatial resolution. While fMRI constrained EEG source imaging seeks to accomplish this task, the static nature of the time-invariant fMRI constraint may unintentionally amplify inaccurate or erroneous results. By creating a time-variant constraint, it is believed that the new algorithm presented here advances current imaging technology by increasing imaging precision and specificity and will thereby enhance our ability to diagnose and treat neural diseases.

## 5. Conclusion

Plasticity manifests in both the physical and functional aspects of the brain. Identifying and understanding the dynamic changes in brain activity that accompany this plasticity stands as one of the major frontiers of biomedical research. Given the limitations in unimodal imaging methods as previously described, a new EEG/fMRI integration method is proposed utilizing fMRI information in a spatially and temporally varying manner to alleviate the sources of error encountered by its predecessor. The performance of the proposed spatiotemporal fMRI constrained source imaging approach was evaluated by comparing against the traditional time-invariant fMRI constrained EEG source imaging in a visual-motor task. Results demonstrated the capability of the proposed approach to noninvasively characterize internal brain activity with high level of spatiotemporal detail. The precision in imaging dynamic brain activity is essential in the study of neuromuscular plasticity mechanisms, characterization of the neuroplastic changes of functional networks in the brain, and evaluation of the progress of cognitive rehabilitation treatments.
